# Correction: Mahmoud et al. Application of Silicon, Zinc, and Zeolite Nanoparticles—A Tool to Enhance Drought Stress Tolerance in Coriander Plants for Better Growth Performance and Productivity. *Plants* 2023, *12*, 2838

**DOI:** 10.3390/plants13030455

**Published:** 2024-02-04

**Authors:** Abdel Wahab M. Mahmoud, Hassan M. Rashad, Sanaa E. A. Esmail, Hameed Alsamadany, Emad A. Abdeldaym

**Affiliations:** 1 Plant Physiology Division, Department of Agricultural Botany, Faculty of Agriculture, Cairo University, Giza 12613, Egypt; mohamed.mahmoud@agr.cu.edu.eg; 2 Department of Biological Sciences, Faculty of Science, King Abdulaziz University, Jeddah 21589, Saudi Arabia; hali@kau.edu.sa (H.M.R.); halsamadani@kau.edu.sa (H.A.); 3 Department of Ornamental Horticulture, Faculty of Agriculture, Cairo University, Giza 12613, Egypt; sanaa.ahmed@agr.cu.edu.eg; 4 Department of Vegetable, Faculty of Agriculture, Cairo University, Giza 12613, Egypt

In the original publication [1], the funding information was not included. The funding are hereby published as follows:

**Funding:** This research was funded by Deanship of scientific research (DSR-KAU) grant No.G:416-130-1443. The authors, therefore thanks DSR for technical and financial support.

The authors state that the scientific conclusions are unaffected. This correction was approved by the Academic Editor. The original publication has also been updated.
